# ﻿Descriptions of two new species of *Phaecadophora* Walsingham, 1900 (Lepidoptera, Tortricidae, Olethreutinae) from China

**DOI:** 10.3897/zookeys.1187.111101

**Published:** 2023-12-21

**Authors:** Yange Li, Wenqing Jing, Shulian Hao, Haili Yu

**Affiliations:** 1 Shaanxi Key Laboratory for Animal Conservation, Northwest University, Xi’an, Shaanxi Province, 710069, China; 2 Tianjin Natural History Museum, Tianjin, 300201, China; 3 Key Laboratory of Resource Biology and Biotechnology in Western China, Northwest University, Ministry of Education, Xi’an, Shaanxi Province, 710069, China

**Keywords:** Olethreutini, *Phaecadophoradactylina* sp. nov., *Phaecadophoravascularis* sp. nov., taxonomy

## Abstract

Two new species of the genus *Phaecadophora*, *P.dactylina***sp. nov.** and *P.vascularis***sp. nov.**, are described from the southwest China. Photographs of the adults and the genitalia are provided. Keys to the species of the genus based on the male and female genitalia are given.

## ﻿Introduction

*Phaecadophora* was proposed by Walsingham (1900) to accommodate two South and East Asian species, *P.fimbriata* Walsingham from Japan, India and Burma, and *P.acutana* Walsingham from Japan. Later, *P.fimbriata* has been reported from China (Meyrick 1914, 1935; Kawabe et al. 1992; Liu and Li 2002; Li et al. 2020), New Guinea (Diakonoff 1953), Vietnam (Kuznetsov 1997), Russia (Kuznetsov 2001), and Thailand (Pinkaew 2007); *P.acutana* has been found in China (Taiwan) (Diakonoff 1973; Kawabe et al. 1992) and Russia (Far East) (Kuznetsov 2001). To date, these two species are the only known members of the genus (Gilligan et al. 2018). In southern China, *P.fimbriata* is widely distributed in 15 provinces. In this paper, we identify two new species from southwestern China (Tibet and Yunnan), and the purpose of this paper is to describe these two species based on morphological features of adults.

## ﻿Materials and methods

The materials examined in this study were collected using light traps. Genitalia dissection followed the methods described by Li (2002). Both adults and genitalia were photographed using a digital microscope (VHX-5000). All specimens used in this study are deposited in the Insect Collection of Northwest University, Xi’an, China (**NWU**).

## ﻿Results

### ﻿ Phaecadophora

Taxon classificationAnimaliaLepidopteraTortricidae

Walsingham, 1900

40A9EC73-6604-5842-A8E5-131CC4E88D7E

Phaecadophora Walsingham, 1900: 130. Type species: Phaecadophorafimbriata Walsingham, 1900.

#### Note.

Walsingham (1900) defined *Phaecadophora* based on external traits, distinguishing it from *Phaecasiophora* Grote by its narrower forewing, densely scaled hind tibia, and the hindwing featuring a long hair-scaled anal margin in males. In fact, the hind tibiae of males in the *Phaecasiophora* species are frequently broadened by long scales and carry one or two long hair pencils. Diakonoff (1973) and Razowski (1989) redescribed the genus, and both asserted its close relationship to *Temnolopha* Lower and *Saliciphaga* Falkovitsh based on the characteristics of both male and female genitalia. However, they did not provide a specific differential diagnosis for *Phaecadophora*. The two previously known species, in conjunction with the two newly described species in this present investigation, exhibit a conspicuous characteristic in their appearance—fine longitudinal stripes on the forewing. They can be defined by the combination of the following genitalia characters: in males, the tegumen is high and narrow; the uncus is slender, hooked, densely spined, and sometimes shortly furcated apically (*P.dactylina* sp. nov.); the socius is small, oval, and densely spined; the gnathos is a simple band, membranous or weakly sclerotized; the valva is robust and curved, often with a deeply concave area (except *P.vascularis* sp. nov.) that separates the sacculus from the densely bristled cucullus; the basal excavation has a protruding rim above which there is a short, apically spined prominence below the costa (*P.dactylina* sp. nov. and *P.vascularis* sp. nov.); the sacculus is weakly spined, occasionally with a tuft of short spine cluster medially on the ventral margin (*P.fimbriata* and *P.dactylina* sp. nov.); the cucullus bears bristles across its base, enlarged and raised on a ridge (*P.fimbriata* and *P.acutana*), and its ventroproximal base is lightly projecting (*P.acutana* and *P.vascularis* sp. nov.) or produced into a ventral process (*P.fimbriata* and *P.dactylina* sp. nov.), which carries a long spine cluster; the caulis is very short; the anellus is a narrow ring; the phallus is short and the cornuti is present (*P.fimbriata*) or absent. In females, the sterigma is derived from a raised fold encircling the ostium and is aciculate; the colliculum is well developed and normal in shape or expanded caudally (*P.fimbriata* and *P.dactylina* sp. nov.); the signa consist of two double-folded plates, either obviously unequal in size (*P.acutana* and *P.vascularis* sp. nov.) or roughly equal in size (*P.fimbriata* and *P.dactylina* sp. nov.).

### ﻿Key to species of *Phaecadophora* based on the male genitalia

**Table:** 

1	Valva with a short prominence above rim of basal excavation below costa	**2**
–	Valva without prominences below costa	**3**
2	Valva constricted beyond basal excavation; sacculus with a short spine cluster on ventral edge at midlength, nearly naked apically; cucullus with basal 1/3 naked except for a tuft of long strong spines and short spines at basal prominence	***P.dactylina* sp. nov.**
–	Valva not constricted beyond basal excavation; sacculus bearing a round patch of fine spines apically just proximal to cucullus, without spine clusters along ventral edge; whole cucullus with dense spines, and carrying a long spine cluster from outer surface of ventral base	***P.vascularis* sp. nov.**
3	Sacculus with a short spine cluster on midlength of ventral edge; cucullus with ventral base expanded and forming a short blunt prominence, apically bearing a strong thorn and a tuft of long spines, these spines longer than uncus	***P.fimbriata***
–	Sacculus with weak spines sparsely along ventral edge; cucullus with ventral base not expanded and carrying a tuft of spines, these spines shorter than uncus	***P.acutana***

### ﻿Key to species of *Phaecadophora* based on the female genitalia

**Table:** 

1	Signa significantly unequal in size	**2**
–	Signa roughly equal in size	**3**
2	Signa oval	***P.acutana***
–	Signa with the large one somewhat broad rectangular, the small one rounded	***P.vascularis* sp. nov.**
3	Sterigma circular, posterior portions not protruding	***P.fimbriata***
–	Sterigma narrow, collar-like, with posterior portion on each side protruding and expanded	***P.dactylina* sp. nov.**

### ﻿ Phaecadophora dactylina

Taxon classificationAnimaliaLepidopteraTortricidae

Li & Yu sp. nov.

E0B73F79-7677-59A0-8831-72C2F333903F

https://zoobank.org/5FDA1766-F739-4A04-8E08-F56DCA536F9E

**Figure 1.**
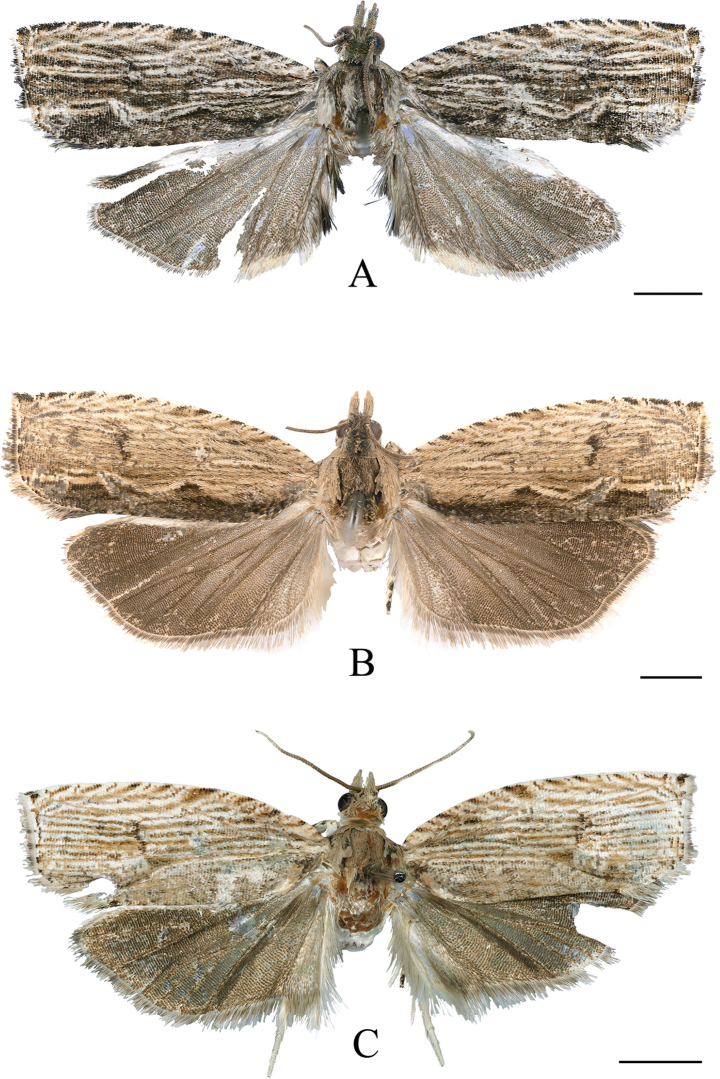
Adults of *Phaecadophora* spp. **A***P.dactylina* sp. nov. (holotype, male) **B***P.dactylina* sp. nov. (paratype, female) **C***P.vascularis* sp. nov. (holotype, male). Scale bars: 2 mm.

**Figure 2.**
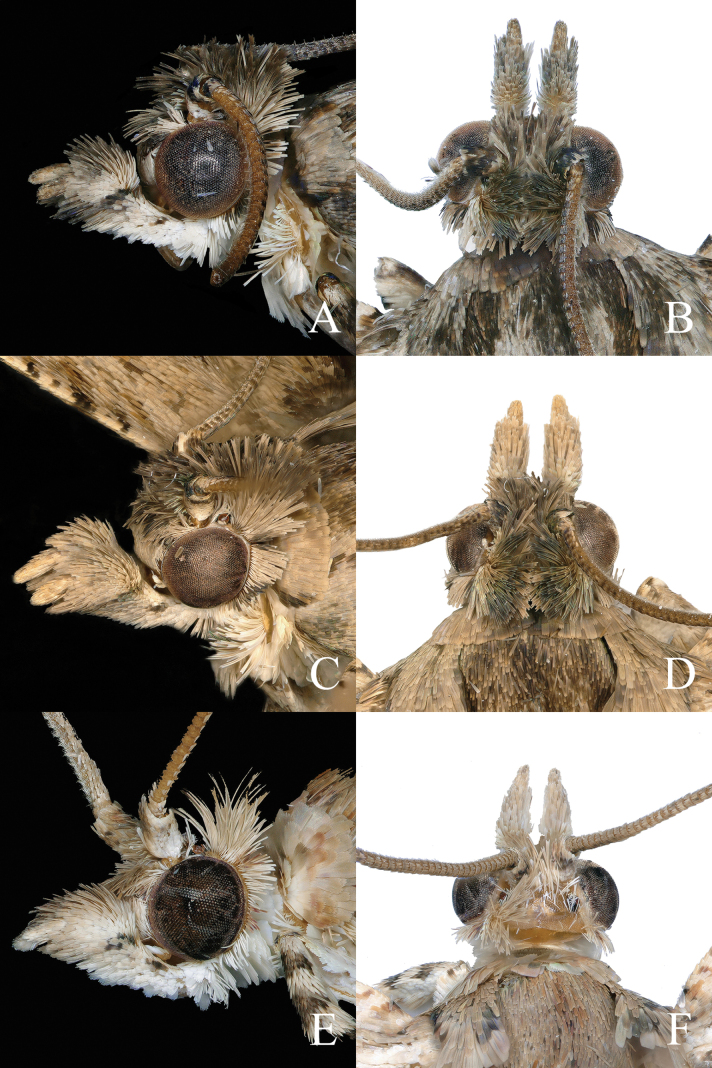
Heads of *Phaecadophora* spp. **A, B***P.dactylina* sp. nov. (holotype, male) **A** lateral view **B** dorsal view **C, D***P.dactylina* sp. nov. (paratype, female) **C** lateral view **D** dorsal view **E, F***P.vascularis* sp. nov. (holotype, male) **E** lateral view **F** dorsal view.

**Figure 3.**
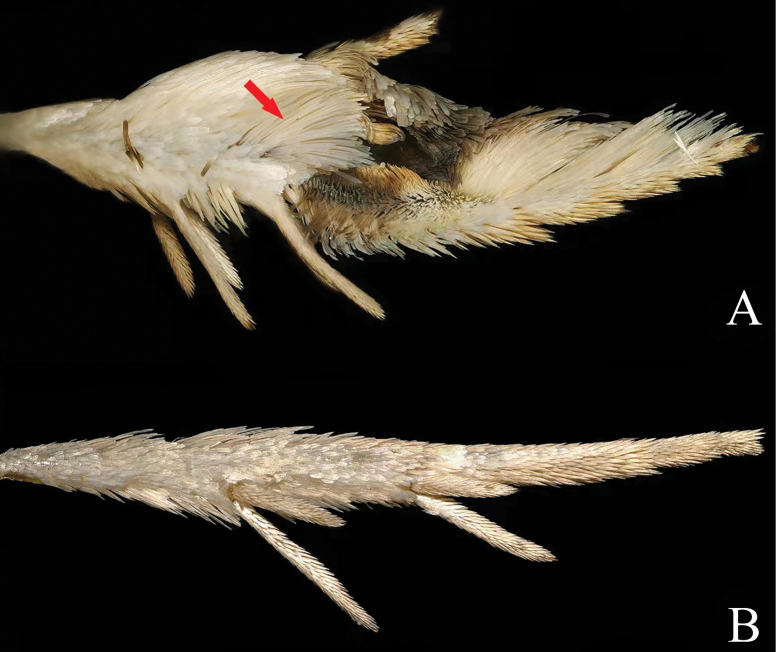
Hind tibiae in male of *Phaecadophora* spp. **A***P.dactylina* sp. nov. (paratype) **B***P.vascularis* sp. nov. (paratype).

**Figure 4.**
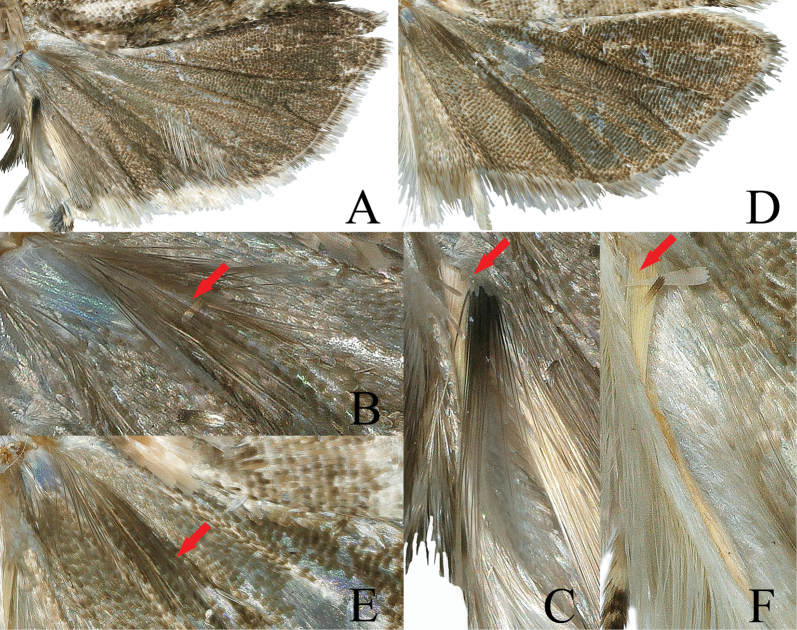
Male hindwings of *Phaecadophora* spp. **A–C***P.dactylina* sp. nov. (paratype) **A** hindwing **B** cubital pecten **C** axillary hair pencils **D–F***P.vascularis* sp. nov. (holotype) **D** hindwing **E** cubital pecten **F** axillary hair pencils.

**Figure 5.**
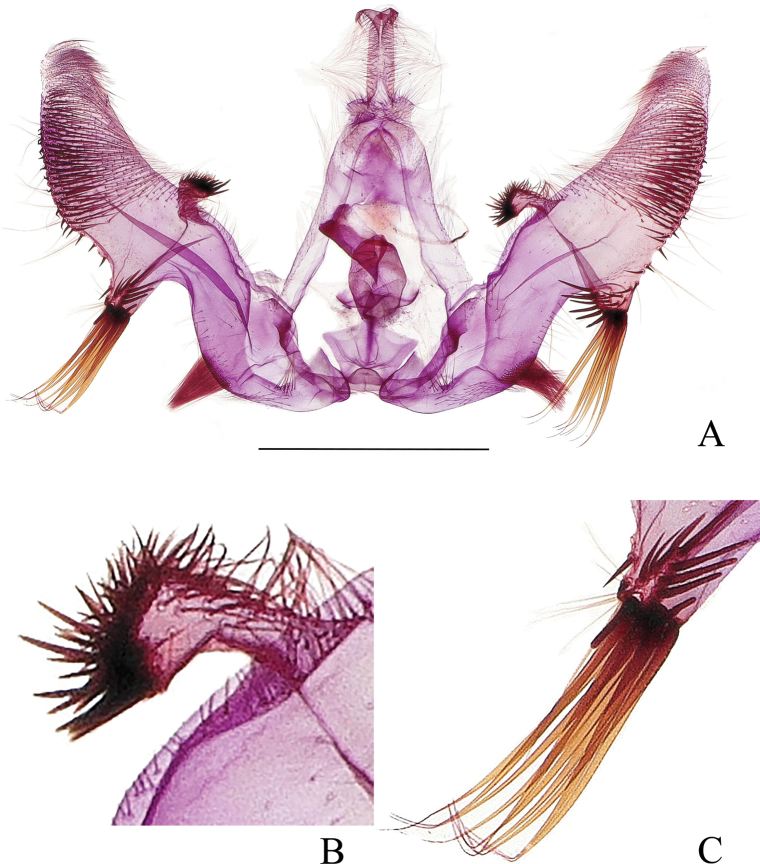
Male genitalia of *Phaecadophoradactylina* sp. nov. (holotype) **A** male genitalia **B** costal prominence of valva **C** ventral prominence of cucullus. Scale bars: 1 mm.

**Figure 6.**
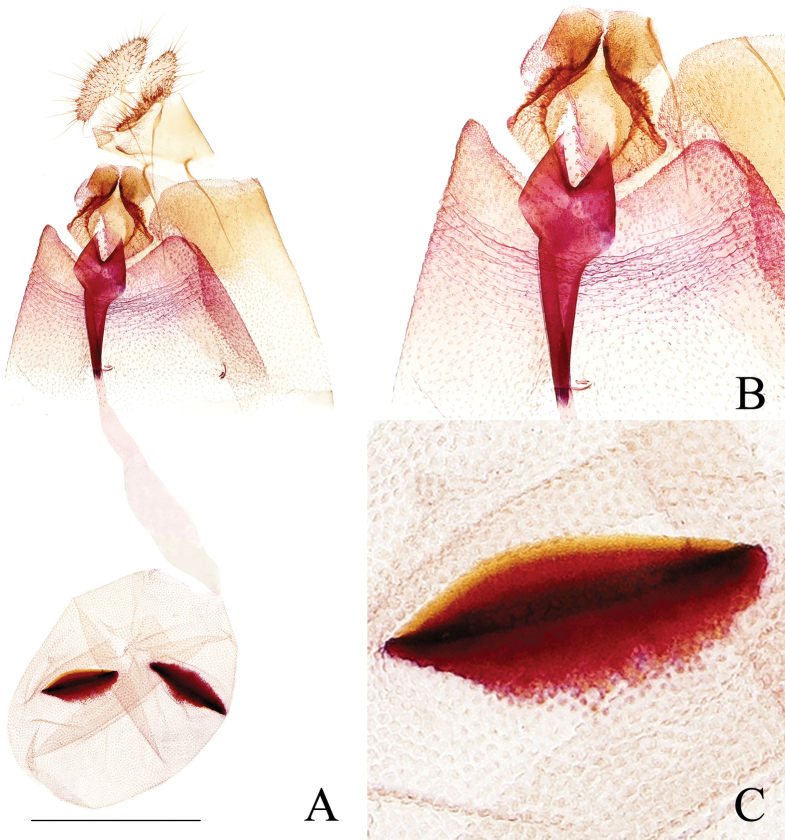
Female genitalia of *Phaecadophoradactylina* sp. nov. (paratype) **A** female genitalia **B** sterigma and colliculum **C** signum. Scale bars: 1 mm.

Figs 1A, B , 2A–D , 3A , 4A–C , 5 , 6

#### Type materials.

***Holotype***: ♂, China, Tibet: Motuo County, Beibengxiang, 29°19.00'N, 95°10.80'E, alt. 810 m, 13 Aug. 2017, Mujie Qi and Xiaofei Yang leg., genitalia slide no. YWX18220. ***Paratypes***: China, Tibet: 3♂, same data as holotype except 29°14.40'N, 95°19.20'E, alt. 810–990 m, 12–13 Aug. 2017; 1♂, same data as holotype except 29°19.20'N, 95°19.20'E, alt. 1100 m, 10 Aug. 2017; 1♀, Nielamu County, 27°58.80'N, 85°58.20'E, alt. 1960 m, 6 Jul. 2019, Mujie Qi and Jiaqi Deng leg.; Yunnan Prov.: 1♂, 3♀, Tengchong County, Linjiapuzi, 25°17.40'N, 98°42.00'E, alt. 2140 m, 15 Aug. 2014, Kaijian Teng, Shurong Liu and Hua Rong leg.

#### Diagnosis.

The male of *P.dactylina* sp. nov. resembles *P.fimbriata* in appearance in having darker scaling in the forewing pattern, two hair pencils and long scales in the anal roll of hindwing, and the hindleg broadened. Dissection of the genitalia is necessary for identification. Conversely, the female can be readily separated from *P.fimbriata* in having the forewing pale brown suffused with tawny longitudinal markings. More diagnostic characters are found in the male and female genitalia. The male of *P.dactylina* sp. nov. can be distinguished by the apically furcated uncus, the valva adorned with a short finger-like prominence below the costa, a nearly bare basal region of the cucullus, and the absence of cornuti in the phallus. In contrast, *P.fimbriata* presents a hooked uncus, the valva devoid of prominences below the costa, and a spined ridge across the base of the cucullus, with the phallus bearing a short spine on the vesica. In the female genitalia, *P.fimbriata* exhibits the sterigma lacking posterior extensions, whereas in *P.dactylina* sp. nov., this structure manifests as two broad plates.

#### Description.

**Male** (Fig. 1A) with forewing length 8.0–9.0 mm. ***Head*** (Fig. 2A, B): vertex and upper frons with shaggy, pale gray-tawny scales (shiny gray distally), lower frons with gray-white appressed scales. Antenna gray-tawny, extending to middle of forewing costa. Ocellus well developed; chaetosema present. Labial palpus ascending, basal half white, distal part gray-white, gray-tawny to gray, medially dusted with a few black scales; median segment broadened distally; terminal segment a little slender, porrect.

***Thorax***: fuscous basally, suffused with gray-white posteriorly. Hind tibia in male short, distally dilated by dense, long scales, creamy white, with a concolorous hair tuft on apical inner surface (Fig. 3A); tarsus strongly broadened by dense scales dorsally; inner side of first segment forming a short, fuscous suffused with brown, scaled cavity; other tarsal segments creamy. Forewing subrectangular, slightly dilated towards termen, costa curved evenly, apex slightly produced, termen weakly oblique, tornus rounded; upperside fuscous, dusted with brown; pairs of strigulae on costa creamy, well-defined striae from them concolorous, extending longitudinally to termen and occupying halfway across the wing, partly confluent below distal half of costa; a double creamy streaks rising from base of wing, zigzagging between cell and 1A+2A to termen above tornus; cilia pale gray on upper part of termen, fuscous on lower part of termen, gray-white on tornus; underside brown, paler on costa, pairs of strigulae creamy, suffused with pale tawny, area of hindwing overlap white. Hindwing (Fig. 4A) fuscous, paler basally, costal area of forewing overlap white; pecten (Fig. 4B) distinct; with a series of long hair-scales between CuA_1_ and distal half of CuA_2_; anal region triangularly expanded, with pale tawny and fuscous long hair pencils (Fig. 4C) in anal roll; anal roll narrowly folded upward, bearing long hair-scales on margin; cilia gray-white; underside brown.

**Female** (Fig. 1B) with forewing length 9.0–10.0 mm. ***Head*** (Fig. 2C, D): vertex and upper frons rough, paler brown; scales shiny gray distally; lower frons with tawny appressed scales. Antenna brown, extending to middle of forewing costa. Ocellus well developed; chaetosema present. Labial palpus ascending, mostly pale brown, paler on inner surface and base; median segment expanded distally, terminal segment porrect, rather slender.

***Thorax***: brown-fuscous. Legs normal. Forewing subrectangular, slightly dilated towards termen, costa curved evenly, termen straight, tornus rounded; upperside with upper 3/4 longitudinally, finely striped with tawny striae from concolorous pairs of costal strigulae and pale brown broken markings, slightly mottled, except a short streak on outer edge of cell; area below 1A+2A and CuA_1_ fuscous, suffused with blackish fuscous, upper edge wavy, produced at middle of fold and base of CuA_1_; cilia fuscous, suffused with brown; underside tawny, pairs of strigulae on costa tawny, area of hindwing overlap white. Hindwing brown-fuscous; costa area of forewing overlap white; pecten distinct; inner side unmodified; cilia pale brown, with brown-fuscous baseline; underside brown.

***Abdomen*: *male genitalia*** (Fig. 5A) with tegumen high and narrow. Uncus slender, densely covered with spines; apex bifurcated, with short spines. Socius small, oval, densely covered with spines. Gnathos membranous, forming a broad band. Valva robust, curved, constricted beyond basal excavation, sacculus nearly half length of valva; a short prominence (Fig. 5B) above the rim of basal excavation below costa, about half of uncus in length, finger-like, apex broadened, with short, dense spines; sacclus well defined, weakly spined, with only sparse, fine hairs beyond basal excavation and along ventral edge, and bearing a spine cluster on midlength of ventral edge; cucullus somewhat elongately triangular, basal 1/3 naked except for strongly protruding ventroproximal base which bears dense, short bristles and a tuft of long bristles apically (Fig. 5C), these long bristles longer than uncus; distal 2/3 of cucullus with dense spines. Phallus short, straight, without cornuti. ***Female genitalia*** (Fig. 6A) with papillae anales narrow, densely setose. Anterior apophysis a little longer than posterior apophysis. Sterigma (Fig. 6B) derived from a raised spinulose fold encircling ostium, with a dorsal notch and posterior portion on each side produced into a broad plate. Colliculum about 1/3 times of length of ductus bursae, strongly sclerotized, caudally wrench-like, abruptly narrowed below, the rest of ductus bursae membranous, ductus seminalis originating posterior to midlength. Corpus bursae ovoid, granulated; signa (Fig. 6C) two, double-folded, roughly equal in size, generally leaf-like.

#### Etymology.

The specific name is derived from the Latin *dactylinus* (= finger-like), referring to the shape of costal prominence of the valva in the male genitalia.

### ﻿ Phaecadophora vascularis

Taxon classificationAnimaliaLepidopteraTortricidae

Li & Yu sp. nov.

A05001E5-0AF0-5977-933A-38CFC2A08745

https://zoobank.org/D963BF72-463D-497F-B0FF-E4B3211E6E67

**Figure 7.**
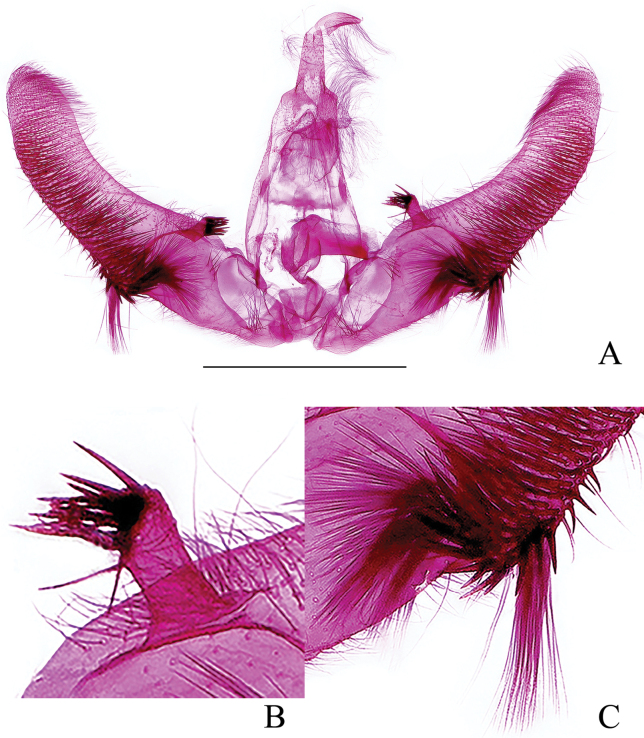
Male genitalia of *Phaecadophoravascularis* sp. nov. (holotype) **A** male genitalia **B** costal prominence of valva **C** ventral base of cucullus. Scale bars: 1 mm.

**Figure 8.**
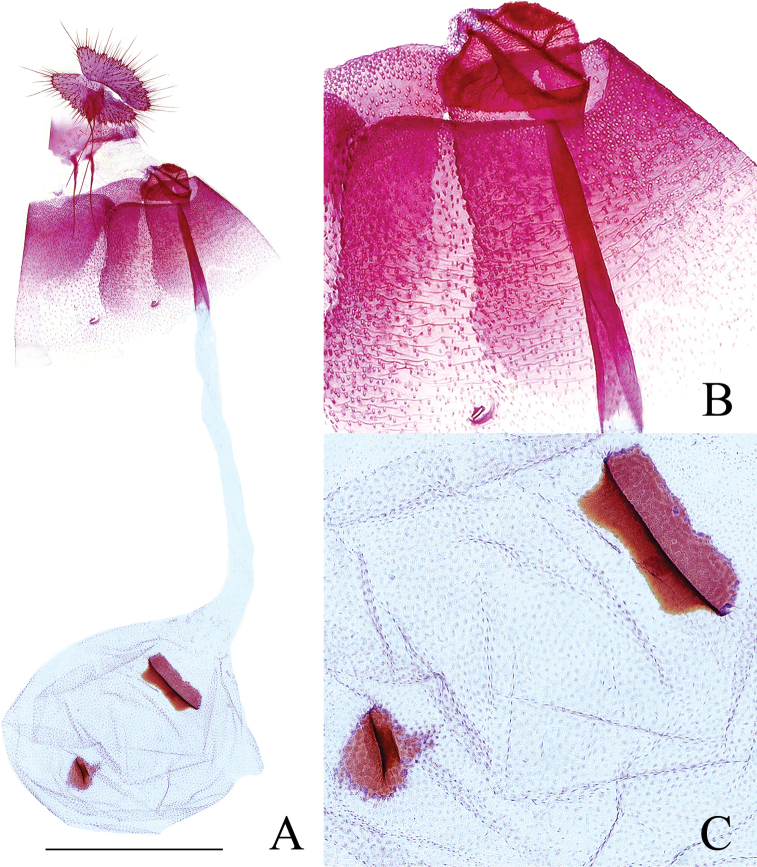
Female genitalia of *Phaecadophoravascularis* sp. nov. (paratype) **A** female genitalia **B** sterigma and colliculum **C** signa. Scale bars: 1 mm.

Figs 1C , 2E, F , 3B , 4D–F , 7 , 8

#### Type materials.

***Holotype***: ♂, China, Yunnan Prov.: Sun River Nature Reserve, 22°36.60'N, 101°06.00'E, alt. 1450 m, 13 May 2014, Zhenguo Zhang leg., genitalia slide no. SXL20569. ***Paratypes***: Yunnan Prov.: 1♂, same data as holotype except 11 May 2014; 1♂, Xishuangbanna Reserve, 21°54.60'N, 101°17.40'E, 21 May 2015, Zhenguo Zhang leg.; 1♀, Tengchong County, Mangbang Town, 25°01.80'N, 98°42.00'E, alt. 1330 m, 10 Aug. 2015, Kaili Liu and Hao Wei leg.

#### Diagnosis.

This species shares similar markings on the forewing with *P.fimbriata* and *P.dactylina* sp. nov., yet its scaling is distinctly pale, particularly dorsal area, which is tawny, suffused with pale brown, as opposed to the fuscous to blackish fuscous hue observed in the latter two species. Furthermore, males of *P.vascularis* sp. nov. has no darkened long scales in the anal roll of the hindwing and instead bear a solitary, pale tawny hair pencil. In contrast, males of *P.fimbriata* and *P.dactylina* sp. nov. present two hair pencils alongside dense, darkened long scales in the anal roll of the hindwing—one hair pencil in pale tawny and the other in blackish fuscous. In the male genitalia, *P.vascularis* sp. nov. exhibits similarities to *P.acutana*; however, it is characterized by the valva featuring a short, finger-like prominence below the base of the costa, a tuft of spines proximal to the base of the cucullus, and the cucullus without a densely spiny transversal ridge basally. While in *P.acutana*, the valva lacks a prominence below the costa, the sacculus bears a tuft of spines under the apical margin, and a densely spiny ridge spans across the base of the cucullus. In the female genitalia, *P.vascularis* sp. nov. can be separated from other species within the genus by possessing two unequal signa, one of which is broadly rectangular, as delineated in the key.

#### Description.

**Adult** (Fig. 1C) with forewing length 8.0–8.5 mm. ***Head*** (Fig. 2E, F): vertex, antenna and upper frons tawny-cream, paler on lower frons; vertex roughly scaled; antenna extending to middle of forewing costa. Ocellus well developed; chaetosema present. Labial palpus ascending, mostly tawny-cream, paler on inner surface and base, medially dusted with gray scales on outer side; median segment distally expanded, terminal segment porrect, rather slender.

***Thorax***: pale tawny, without posterior crest. Hind tibia white, simple, not modified in male, without hair pencils (Fig. 3B). Forewing subrectangular, costa curved basally and nearly straight distally, termen vertical and straight, tornus rounded; upper side with complex pattern of distinct longitudinal, fine, parallel creamy and pale brown lines, dusted with fuscous rising from base and costa to termen, interrupted by a short, transverse, fuscous marking on outer edge of cell; cilia tawny-white, white on tornus; underside brown, costa pale brown, with pairs of strigulae creamy, area of hindwing overlap white. Hindwing (Fig. 4D) fuscous except costal area of forewing white; cubital pecten (Fig. 4E) present; in male anal region expanded, with anal roll bearing a long pencil (Fig. 4F) of pale tawny hair-scales from base of wing; cilia pale brown basally and pale white apically; underside brown.

***Abdomen*: *male genitalia*** (Fig. 7A) with tegumen high and narrow, shoulders obvious. Uncus a slender hook, densely spined. Socius small, oval, densely spined. Gnathos membranous. Valva moderate in width, curved, without neck; sacculus about 1/3 times of length of valva, its ventral edge nearly straight; a short finger-like prominence (Fig. 7B) below base of costa and above the protruding rim of basal excavation, with dense, short spines apically; sacculus with sparse spines basally and a rounded tuft of spines proximal to cucullus; cucullus with dense spines and a slender spine cluster (Fig. 7C) under its ventroproximal base, with these spines shorter than uncus. Phallus short, without cornuti. ***Female genitalia*** (Fig. 8A) with papillae anales narrow, densely setose. Anterior apophyses a little shorter than posterior apophyses. Sterigma (Fig. 8B) a finely spinulose, inverted, blunt triangular area with a median split containing ostium. Colliculum moderately sclerotized, about 1/4 times of ductus bursae in length, inception of ductus seminalis posterior to midlength. Corpus bursae ovoid, granulated; signa (Fig. 8C) two, double-folded, unequal in size, the large one like a broad and shallow basket, somewhat rectangular, the small one a little oval, about half of the larger one in size.

#### Etymology.

The specific name is derived from the Latin *vascularis* (= veined), referring to the markings of forewing.

## ﻿Discussion

Diakonoff (1973) classified *Phaecadophora* in the subtribe Neopotamiae based on the shape of signa. Most members of Neopotamiae are characterized by relatively large adults, as does *Phaecadophora*, whose forewing ranges from 6.0 mm to 9.5 mm in length. Distinguishing features include the presence of slender longitudinal stripes on the forewing from the base to the termen, setting adults of *Phaecadophora* apart from most genera in Olethreutini, where a common forewing pattern involves several dark, parallel, outwardly oblique, transverse bands—albeit with various developments and modifications. *Phaecadophora* species, especially those discussed here and *P.fimbriata*, share considerable external similarities. However, polymorphism in forewing patterns is frequently observed in adults of *Phaecadophora*. For instance, specimens from Japan exhibit at least five types of forewing patterns in *P.acutana*, (three in females and two in males) (Nasu 2013) and two types in females of *P.fimbriata*. In this study, sexual dimorphism is noted in *P.dactylina* sp. nov. Although the morphological polymorphism of *P.vascularis* sp. nov. remains inconclusive due to a limited sample size (only four specimens were observed and collected in close proximity), it is crucial to exercise caution in drawing conclusions. Specimens of *P.fimbriata* from China present a perplexing case, displaying a constant darkened forewing pattern (one of two types in Japanese specimens) across a broad geographical range (between 17–31°N and 97–120°E), without observed dimorphism or polymorphism. Despite marked differences in scent organs among the males of these four species, intriguingly, they exhibit similarities in pairs. Males of *P.fimbriata* and *P.dactylina* sp. nov. demonstrate complex modifications in the hindleg, with the tibia and tarsus strongly broadened, laterally compressed, and forming a cavity (Fig. 3A). They also exhibit modifications in the hindwing, featuring two hair pencils and long scales in the anal roll (Fig. 4C), as well as in the abdomen, with two median tufts of appressed scaling on sternites V–VII. On the other hand, males of *P.acutana* and *P.vascularis* sp. nov. lack modifications in the hindleg (Fig. 3B) and abdomen, carrying only one hair pencil in the anal roll of the hindwing (Fig. 4F). Notably, scent organs prove challenging for classification, as acknowledged by Diakonoff (1981a) and Aarvik (2004). Generally, dissection of the genitalia is necessary for reliable identification. In males, the valva provides optimal diagnostic characters, while in females, the specific distinction lies in the shapes of the sterigma and signa.

Diakonoff (1973, 1981b), Razowski (1989), and Horak (2006) extensively elucidated the close relationship between *Phaecadophora*, *Saliciphaga*, and *Temnolopha*. In comparison to *Temnolopha*, a suite of genital characteristics in *Phaecadophora* suggests a more intimate connection with *Saliciphaga*. These features include a high, triangular tegumen, a somewhat hooked uncus that is slender, a small oval socius, symmetrical valvae with relatively narrow sacculus, and two large, double-folded signa. While the two previously known species, *P.fimbriata* and *P.acutana*, lack a basal valval prominence in the male genitalia, this structure is well developed in the two species described here, referring the genus to vicinity of *Neopotamia* Diakonoff, 1973. The dorsally split and dorsocaudally enlarged sterigma, along with well-defined signa lacking basal scobination, further supports this association.

## Supplementary Material

XML Treatment for Phaecadophora

XML Treatment for Phaecadophora dactylina

XML Treatment for Phaecadophora vascularis

## ﻿Additional information

### Author ORCIDs

Yange Li https://orcid.org/0009-0002-9188-2178

Wenqing Jing https://orcid.org/0009-0007-9306-8854

Shulian Hao https://orcid.org/0009-0005-6556-4151

Haili Yu https://orcid.org/0000-0003-1249-5461

### Data availability

All of the data that support the findings of this study are available in the main text.
